# Review of Bats and SARS

**DOI:** 10.3201/eid1212.060401

**Published:** 2006-12

**Authors:** Lin-Fa Wang, Zhengli Shi, Shuyi Zhang, Hume Field, Peter Daszak, Bryan T. Eaton

**Affiliations:** * Australian Animal Health Laboratory, Geelong, Victoria, Australia;; † Wuhan Institute of Virology of Chinese Academy of Sciences, Wuhan, People’s Republic of China;; ‡ Institute of Zoology of Chinese Academy of Sciences, Beijing, People’s Republic of China;; § East China Normal University, Shanghai, People’s Republic of China; ¶ Department of Primary Industries and Fisheries, Brisbane, Queensland, Australia;; # Consortium for Conservation Medicine, New York, New York USA

**Keywords:** emerging zoonoses, SARS, coronavirus, bats, animal reservoir, spillover, synopsis

## Abstract

Bats have been identified as a natural reservoir for an increasing number of emerging zoonotic viruses, including henipaviruses and variants of rabies viruses. Recently, we and another group independently identified several horseshoe bat species (genus *Rhinolophus*) as the reservoir host for a large number of viruses that have a close genetic relationship with the coronavirus associated with severe acute respiratory syndrome (SARS). Our current research focused on the identification of the reservoir species for the progenitor virus of the SARS coronaviruses responsible for outbreaks during 2002–2003 and 2003–2004. In addition to SARS-like coronaviruses, many other novel bat coronaviruses, which belong to groups 1 and 2 of the 3 existing coronavirus groups, have been detected by PCR. The discovery of bat SARS-like coronaviruses and the great genetic diversity of coronaviruses in bats have shed new light on the origin and transmission of SARS coronaviruses.

## Abstract 2

TOC Summary: The discovery of SARS-like coronaviruses in horseshoe bats highlights the possibility of future outbreaks caused by different coronaviruses of bat origin.

Severe acute respiratory syndrome (SARS) represents the 21st century's first pandemic of a transmissible disease with a previously unknown cause. The pandemic started in November 2002 and was brought under control in July 2003, after it had spread to 33 countries on 5 continents, resulting in >8,000 infections and >700 deaths (*1*). The outbreaks were caused by a newly emerged coronavirus, now known as the SARS coronavirus (SARS-CoV).

In late 2003 and early 2004, sporadic outbreaks were reported in the region of the People's Republic of China where the 2002–2003 outbreaks originated (*2*). However, molecular epidemiologic studies showed that the viruses responsible for the 2003–2004 outbreaks were not the same as those isolated during the 2002–2003 outbreaks (*3*). These findings indicate independent species-crossing events. They also indicate that a SARS epidemic may recur in the future and that SARS-like coronaviruses (SARS-like–CoVs) that originate from different reservoir host populations may lead to epidemics at different times or in different regions, depending on the distribution of the reservoirs and transmitting hosts. The recent discovery of a group of diverse SARS-like–CoVs in bats supports the possibility of these events and further highlights the need to understand reservoir distribution and transmission to prevent future outbreaks.

## Animal Origin of SARS Coronaviruses

Because of the sudden and unpredictable nature of the SARS outbreaks that started in November 2002 in southern People's Republic of China, structured and reliable epidemiologic studies to conclusively trace the origin of SARS-CoV were not conducted. However, accumulated studies from different groups, which used a variety of approaches, indicated an animal origin on the basis of the following findings. 1) Genome sequencing indicated that SARS-CoV is a new virus with no genetic relatedness to any known human coronaviruses (*4**,**5*). 2) Retrospective serologic studies found no evidence of seroprevalence to SARS-CoV or related viruses in the human population (*6*). 3) Serologic surveys among market traders during the 2002–2003 outbreaks showed that antibodies against SARS-CoV or related viruses were present at a higher ratio in animal traders than control populations (*7**–**9*). 4) Epidemiologic studies indicated that early case-patients were more likely than later case-patients to report living near a produce market but not near a farm, and almost half of them were food handlers with probable animal contact (*7*). 5) SARS-CoVs isolated from animals in markets were almost identical to human isolates (*9*). 6) Molecular epidemiologic analyses indicated that human SARS-CoV isolates could be divided into 3 groups from the early, middle, and late phases of the outbreaks and that early-phase isolates were more closely related to the animal isolates (*10*). 7) Human SARS-CoVs isolates from the 2003–2004 outbreaks had higher sequence identity to animal isolates of the same period than to human isolates from the 2002–2003 outbreaks (*3*).

## Susceptible Animals in Markets and Laboratories

The first evidence of SARS-CoV infection in animals came from a study conducted in a live animal market in early 2003 (*9*). From the 25 animals sampled, viruses closely related to SARS-CoV were detected in 3 masked palm civets (Paguma larvata) and 1 raccoon dog (Nyctereutes procyonoides). In addition, neutralizing antibodies against SARS-CoV were detected in 2 Chinese ferret badgers (Melogale moschata). This initial study indicated that at least 3 different animal species in the Shenzhen market were infected by coronaviruses that are closely related to SARS-CoV.

Given the vast number of live animals being traded in animal markets in southern People's Republic of China, knowing which other animals are also susceptible to these viruses is crucial. Unfortunately, for a variety of reasons no systematic studies were conducted on traded animals during the outbreak period. Experimental infection of different animals therefore became a component of the SARS-CoV investigation.

Currently, >10 mammalian species have been proven to be susceptible to infection by SARS-CoV or related viruses (Table 1). Rats were also implicated as potentially susceptible animals that may have played a role in the transmission and spread of SARS-CoV in the well-publicized SARS outbreaks in the Amoy Gardens apartment block in Hong Kong Special Administrative Region, People's Republic of China (*23*). In Guangdong in 2004, the first human with a confirmed case of SARS was reported to have had no contact with any animals except rats (*2*). Experimentally, we have obtained serologic evidence that SARS-CoV replicates asymptomatically in rats (B.T. Eaton et al., unpub. data). Further studies are needed to clarify the potential role of rats in the transmission of SARS-CoV. Studies by 2 independent groups suggested that avian species were not susceptible to SARS-CoV infection and that, hence, domestic poultry were unlikely to be the reservoir or associated with the dissemination of SARS-CoV in the animal markets of southern People's Republic of China (*22**,**24*).

**Table 1:** Animal species susceptible to infection by SARS coronavirus*

Animal		Mode of infection	Clinical signs	References
Common name	Taxonomic name			
Masked palm civet	*Paguma larvata*	Natural	None observed	(*9*)
		Experimental	Fever, lethargy, reduced appetite	(*11*)
Racoon dog	*Nyctereutes procyonoides*	Natural	None observed	(*9*)
Chinese ferret badger	*Melogale moschata*	Natural	None observed	(*9*)
Cynomolgus macaque	*Macaca facicularis*	Experimental	Lethargy, skin rash, respiratory distress	(*12*)
Rhesus macaque	*Macaca mulatta*	Experimental	Fever, low appetite	(*13**,**14*)
African green monkey	*Cercopithecus aethiops*	Experimental	None observed	(*15*)
Ferret	*Mustela furo*	Experimental	Lethargy, mild pulmonary lesions	(*16*)
Golden hamster	*Mesocricetus auratus*	Experimental	None observed	(*17*)
Guinea pig	*Cavia porcellus*	Experimental	None observed	(*18*)
Mouse	*Mus musculus*	Experimental	Aged animal (12–14 mo): weight loss, hunched posture, ruffled fur, slight dehydration	(*19*)
			Young animal (4–6 weeks): none observed	(*20*)
Rat	*Rattus rattus*	Experimental	None observed	B.T. Eaton et al., unpub. data
Domestic cat	*Felis domesticus*	Natural	Not reported	(*16*)
		Experimental	None observed	(*16*)
Pig	*Sus scrofa*	Natural	Not reported	(*21*)
		Experimental	None observed	(*22*)

*SARS, severe acute respiratory syndrome.

## Role of Masked Palm Civets

Although in 1 live animal market, 3 species were found to be infected by viruses related to SARS-CoV (*9*), all subsequent studies have focused mainly on palm civets, possibly because the rate of detection was higher in civets or because the number of civets traded in southern People's Republic of China exceeds that of other wildlife groups.

The isolation of closely related SARS-CoV in civets during the 2002–2003 and 2003–2004 outbreaks and the close match of virus sequences between the human and civet isolates from each outbreak (*3**,**9**,**25*) strongly suggest that civets are a direct source of human infection. However, these studies did not clarify whether animals other than civets were involved in transmission of SARS-CoV to humans or whether civets were an intermediate host or the natural reservoir host of SARS-CoVs.

During the 2002–2003 outbreaks, none of the animal traders surveyed in the markets, who supposedly had very close contact with live civets, displayed SARS symptoms (*7**–**9*). During the 2003–2004 outbreaks, at least 1 human SARS patient had had no contact with civets (*2*). These observations seem to indicate that >1 other animal species may play a role in transmission of SARS-CoV to humans.

Most, if not all, civets traded in the markets are not truly wildlife animals; rather, they are farmed animals. Civet farming is relatively new in People's Republic of China and has rapidly expanded during the past 15 years or so. Tu et al. conducted the first comparative study of market and farmed civets (*26*). Serologic testing was performed on 103 serum samples taken from civets in an animal market in Guangdong and several civet farms in different regions of People's Republic of China in June 2003 and January 2004. No significant level of SARS-CoV antibody was detected in any of the 75 samples taken from 6 farms in 3 provinces. In contrast, of the 18 samples taken from an animal market in Guangdong Province in January 2004, 14 (79%) had neutralizing antibodies to SARS-CoV.

In a parallel study conducted between January and September 2004 (*27*), molecular analysis was used to investigate the distribution of SARS-CoV in palm civets in markets and on farms. PCR analysis of samples from 91 palm civets and 15 raccoon dogs in 1 animal market and 1,107 civets from 25 farms in 12 provinces showed positive results for all animals from the market and negative results for all animals from the farms. Similar results were obtained in wild-trapped civets in Hong Kong; none of the 21 wild civets sampled had positive antibody or PCR results for SARS-CoV (*28*).

Although not universally true, natural reservoir hosts tend to have coevolved with their viruses and usually do not display clinical signs of infection (*29*). However, when palm civets were experimentally infected with 2 strains of human SARS-CoV, all developed clinical signs of fever, lethargy, and loss of aggressiveness (*11*).

Civits' high susceptibility to SARS-CoV infection and wide presence in markets and restaurants strongly indicates an important role for civets in the 2002–2003 and 2003–2004 SARS outbreaks. However, the lack of widespread infection in wild or farmed palm civets makes them unlikely to have been the natural reservoir host.

## SARS-like Coronaviruses in Bats

The presence of SARS-like–CoVs in different species of horseshoe bats in the genus Rhinolophus has recently been reported. We found, in a study of horseshoe bat species in different regions of mainland People's Republic of China in 2004 (*30*), that each of the 4 species surveyed had evidence of infection by a SARS-like–CoV: 2 species (R. pearsoni and R. macrotis) had positive results by both serologic and PCR tests, and 2 (R. pussilus and R. ferrumequinum) had positive results by either serologic or PCR tests, respectively. Bats with positive results were detected in the provinces of Hubei and Guangxi, which are >1,000 km apart. A group in Hong Kong (*31*) found that, when analyzed by PCR, 23 (39%) of 59 anal swabs of wild Chinese horseshoe bats (R. sinicus) contained genetic material closely related to SARS-CoV. They also found that as many as 84% of the horseshoe bats examined contained antibodies to a recombinant N protein of SARS-CoV. A previous study indicated a certain level of antigenic cross-reactivity between SARS-CoV and some group 1 coronaviruses (*6*) and that several group 1 coronaviruses had recently been found in bats. Therefore, the actual seropositive proportion of R. sinicus might be <84%. Nevertheless, the relatively high seroprevalence and wide distribution of seropositive bats is consistent with the serologic pattern expected from a pathogen's natural reservoir host (*29*).

Genome sequencing showed that the genome organization of all bat SARS-like–CoVs is almost identical to that of the SARS-CoVs isolated from humans or civets. They shared an overall sequence identity of 88% to 92%. The most variable regions were located in the 5´ end of the S gene, which codes for the S1 domain responsible for receptor binding, and in open reading frame 10 (ORF10 or ORF8, depending on the nomenclature used) region immediately upstream from the N gene (Figure, panel A, region b), which is known to be also prone to deletions of various sizes (*3**,**9*). Most human SARS-CoVs isolated during the late phase of the 2002–2003 outbreaks have a 29-nt deletion in this region; this deletion is absent in the civet isolates or human isolates from the early phase of the outbreaks (*3**,**9*). The bat viruses also lack the 29-nt deletion, indicating that SARS-CoVs and SARS-like–CoVs share a common ancestor.

**Figure:**
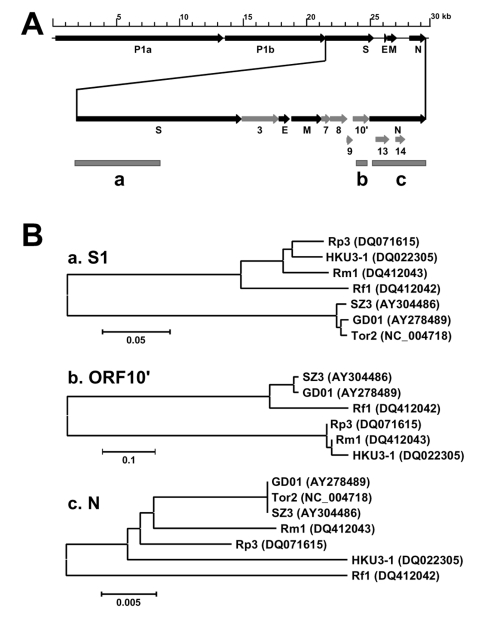
A) Genome diagram indicating the location of structural (dark arrow) and nonstructural (shaded arrow) genes and the different regions (shaded boxes) used for phylogeny analysis. B) Phylogenetic trees based on deduced amino acid sequences of the spike protein S1 domain (a), the open reading frame (ORF)10' (b), and the N protein (c). Because of lack of the ORF10' coding region in Tor2, Tor2 could not be included for the tree in (b). GD01, human isolate from early phase of the outbreak in 2003; Tor2, human isolate from late phase of the outbreak in 2003; SZ3, civet isolate from March 2003; Rp3, bat isolate from Rhinolophus pearsoni, December 2004; Rf1, bat isolate from R. ferrumequinum, November 2004; Rm1, bat isolate from R. macrotis, November 2004; and HKU3–1, bat isolate from R. sinicus, February 2005. GenBank accession nos. appear next to isolate names.

Furthermore, sequence analyses indicated the existence of a much greater genetic diversity of SARS-like–CoVs in bats than of SARS-CoVs in civets or humans, which supports the notion that SARS-CoV is a member of this novel coronavirus group and that bats are a natural reservoir for it. The overall genome sequence identities between the human/civit SARS-CoVs and the bat viruses Rp3 (isolated from R. pearsoni) and HKU3–1 (isolated from R. sinicus) were 92% and 88%, respectively. The sequence identity between the bat isolates Rp3 and HKU3–1 is 89%, which indicates that the genetic divergence among the bat isolates is as great as the divergence between each of the bat viruses and the human/civet isolates. In addition, phylogenetic trees based on different protein sequences display different tree topologies, as shown in the Figure (panel B), which indicates the existence of multiple evolutionary pathways for different regions of the genome.

### Diversity of Coronaviruses in Bats

The discovery of SARS-CoV has boosted the search for novel coronaviruses of human and animal origin. Bats have been chosen as the main target because of their species diversity, large population size, broad geographic distribution, ability for long-distance migration, and habit of roosting in large groups. In addition to the SARS-like–CoVs described above, many other coronaviruses have been detected by PCR among diverse bat populations in Hong Kong (Table 2).

**Table 2:** Coronaviruses detected in different species of bats

Viruses		Bat species	Location of detection (People’s Republic of China)	Reference
Group*	Name/strain			
G1	Bat-CoV HKU2	*Rhinolophus sinicus*	Hong Kong	(*32*)
	Bat-CoV HKU6	*Myotis ricketti*	Hong Kong	(*32*)
	Bat-CoV HKU7	*Miniopterus magnater*	Hong Kong	(*32*)
	Bat-CoV HKU8	*Miniopterus pusillus* *M. magnater* *Miniopterus schreibersii*	Hong Kong	(*28**,**32*)
	BtCoV/701/05	*Myotis ricketti*	Anhui, Yunnan, Guangdong	(*33*)
	BtCoV/821/05	*Myotis ricketti*	Jiangxi, Guangxi	(*33*)
	BtCOV/821/05	*Scolophlus kuhlii*	Hainan	(*33*)
	BtCoV/970/06	*Rhinolophus pearsoni* *Rhinolophus ferrumequinum*	Shandong	(*33*)
	BtCoV/A773/05	*M. schreibersii*	Fujian	(*33*)
	BtCoV/A011/05	*M. schreibersii*	Anhui, Fujian, Guangxi	(*33*)
G2b	Rp3	*R. pearsoni*	Guangxi	(*30*)
	Rm1 (BtCoV/279/04)	*Rhinolophus macrotis*	Hubei	(*30*)
	Rf1 (BtCoV/273/04)	*R. ferrumequinum*	Hubei	(*30*)
	Bat-SARS-CoV HKU3	*R. sinicus*	Hong Kong	(*31**,**32*)
	BtCoV/A1018/06	*R. sinicus*	Shandong	(*33*)
	BtCoV/279/04	*R, macrotis*	Hubei	(*33*)
	BtCoV/273/04	*R. ferrumequinum*	Hubei	(*33*)
G2c	Bat-CoV HKU4	*Tylonycteris pachypus*	Hong Kong	(*32*)
	Bat-CoV HKU5	*Pipistrellus abramus*	Hong Kong	(*32*)
	BtCoV/133/05	*T. pachypus*	Guangdong	(*33*)
	BtCoV/434/05	*Pipistrellus pipistrellus*	Hainan	(*33*)
	BtCoV/355/05	*P. abramus* *R. ferrumequinum*	Anhui, Henan, Sichuan	(*33*)

*Group 2 was subdivided as in (*32*). A different classification was used in (*33*), in which G2b and G2c were designated G4 and G5, respectively.

Poon et al. (*28*) conducted a surveillance study in Hong Kong during the summers of 2003 and 2004. From 162 swab samples collected from 12 bat species, they detected a novel group 1 coronavirus by sequencing of PCR products from the RNA-dependent RNA polymerase (RdRp) gene. The same virus or viruses of the same genetic lineage were found in 3 Miniopterus species (M. pusillus, M. magnater, and M. schreibersii). However, attempts to isolate virus by using 3 different cell lines (MDCK, FRhK4, and VeroE6) were unsuccessful.

In another study in Hong Kong during April 2004–July 2005, Woo et al. (*32*) sampled 309 individual bats representing 13 species from 20 different locations in rural Hong Kong. They detected coronavirus-related viral genomic RNA in 37 bats, representing a prevalence of 12%. Partial sequencing of RdRp and helicase genes identified 8 coronavirus genome types, 2 of which were the same as those reported previously (*28**,**30**,**31*). The other 6 novel types of coronaviruses were obtained from 6 different bat species and phylogenetically positioned in 2 of the existing 3 coronavirus groups. Four were in group 1, derived from bat species M. magnater, M. pusillus, Myotis ricketti, and R. sinicus; the other 2 were in group 2, from bat species Pipistrellus abramus and Tylonycteris pachypus. To accommodate the newly discovered genetic diversity of group 2 coronaviruses, the authors proposed the following subdivisions: group 2a (coronaviruses existing before the discovery of SARS-CoV), group 2b (SARS-CoV and SARS-like–CoVs), and group 2c (novel bat coronaviruses discovered in this study). Attempts to isolate virus in VeroE6, MRC-5, LLC-Mk2, FRhK-4, Huh-7.5, and HRT-18 were unsuccessful.

In another extensive study conducted in mainland People's Republic of China during November 2004–March 2006, Tang et al. (*33*) collected samples from 985 bats: 35 species in 14 genera and 3 families at 82 different sites in 15 provinces. A total of 64 (6.5%) bats had positive results from a PCR directed to a highly conserved 440-bp RdRp region. Among the 64 PCR-positive products sequenced, only 3 (all from the genus Rhinolophus) were clustered with known bat SARS-like–CoVs (or group 2b), 40 belonged to group 1, and the remaining 22 formed a separate cluster in group 2, most likely clustering with the group 2c viruses reported by Woo et al. (*32*). Attempts to isolate virus in VeroE6, FRHK4, and CV1 were unsuccessful.

In addition to the diversity of coronaviruses in bats, 3 more observations can be drawn from these studies. First, none of the bat coronaviruses discovered so far belonged to group 3. Second, with very few exceptions, most bat coronaviruses seem to be species-specific; i.e., different bat species from a similar location harbor different coronaviruses, whereas the same bat species from different geographic locations carry coronaviruses of the same genetic lineage (*32**,**33*). Third, among the 5 published studies involving bat coronaviruses (*28**,**30**–**33*), no researchers were able to isolate live virus from any of the swab samples collected despite the use of many different cell lines and the presence of high levels of viral genetic materials shown by quantitative PCR.

### Cross-species Transmission

Emergence of zoonotic viruses from a wildlife reservoir requires 4 events: 1) interspecies contact, 2) cross-species virus transmission (i.e., spillover), 3) sustained transmission, and 4) virus adaptation within the spillover species (*34*). These 4 transition events occurred during the SARS outbreaks and contributed to the rapid spread of the disease around the world.

The role of civets in directly transmitting SARS-CoV to humans has been well established. The most convincing case was the infection of a waitress and a customer in a restaurant where SARS-CoV–positive civets were housed in cages (*25*). Two key questions remain: What is the natural reservoir host for the outbreak SARS-CoV strains, and how were the viruses transmitted to civets or other intermediate hosts? Although not conclusive, the data obtained so far strongly suggest that bats (horseshoe bats in particular) are most likely the reservoir host of SARS-CoV. As indicated above, bat coronaviruses seem to be species-specific and SARS-like–CoVs discovered so far are exclusively associated with horseshoe bats. We hope that continued field study will eventually identify the direct progenitor of SARS-CoV among the 69 different known horseshoe species. The facts that the cross-species transmission of SARS-CoV seems to be a relatively rare event and that legal and illegal trading of wildlife animals between People's Republic of China and other countries occurs raise the possibility that the natural reservoir species may not be native to People's Republic of China. Thus, we should expand our search into regions other than Hong Kong and mainland People's Republic of China. Another approach to search for the natural reservoir of SARS-CoV is to conduct infection experiments in different bat species. If we assume that the progenitor viruses come from bats, chances are high that the human/civet SARS-CoVs are still capable of infecting the original reservoir species.

Without knowing the natural reservoir of SARS-CoV, predicting the exact mechanism of transmission from reservoir host to intermediate host is difficult. However, the fecal-oral route represents the main mode of transmission among animals. Although mixing of live reservoir hosts (e.g., bats) and intermediate hosts (e.g., civets) would be an efficient means of transmission, the main source of cross-species transmission in the animal trading chain (including warehouses, transportation vehicles, markets) may come from contaminated feces, urine, blood, or aerosols. This may also be true for civet-to-human transmission. As shown in the case of the infected restaurant customer in 2004, the customer had no direct contact with civets and was sitting at a table ≈5 m from the civet cages (*25*).

Although at this stage we cannot rule out the possibility of direct transmission from the natural reservoir host to humans, molecular epidemiologic studies (*2**,**10*) and studies of the receptor-S protein interaction (*35*) indicate that the progenitor viruses are unlikely to be able to infect humans and that a rapid viral evolution in an intermediate host (such as civets) seems to be necessary to adapt the virus for human infection. Ability to efficiently use the receptor molecules (ACE2 for human and civet) seems to be a major limiting factor for animal-to-human and human-to-human transmission (*35*). This also explains why the SARS-CoV was able to cause the human pandemic but the closely related bat SARS-like–CoVs were not. For the SARS-like–CoVs to infect humans, substantial genetic changes in the S1 receptor-binding domain will be necessary. These changes may be achieved in 1 of 2 possible ways. They could be achieved by genetic recombination, as coronaviruses are known to be able to recombine. For example, bat SARS-like–CoVs and another yet unknown coronavirus could coinfect an intermediate host, and the bat viruses would gain the ACE2 binding site in the S1 domain by recombination. The alternative is continuous evolution independent of recombination. Coronaviruses in bats could have a spectrum sufficiently diverse to encompass the progenitor virus for SARS-CoVs. The progenitor virus's ability to bind human ACE2 may be acquired or improved by adaptation (i.e., point mutations) in >1 intermediate host before it could efficiently infect humans. The existence of at least 3 discontinuous highly variable genomic regions between SARS-CoV and SARS-like–CoV indicates that the second mechanism is more likely.

In conclusion, the discovery of bat SARS-like–CoVs and the great genetic diversity of coronaviruses in bats have shed new light on the origin and transmission of SARS-CoV. Although the exact natural reservoir host for the progenitor virus of SARS-CoV is still unknown, we believe that a continued search in different bat populations in People's Republic of China and neighboring countries, combined with experimental infection of different bat species with SARS-CoV, will eventually identify the native reservoir species. A positive outcome of these investigations will greatly enhance our understanding of spillover mechanisms, which will in turn facilitate development and implementation of effective prevention strategies. The discovery of SARS-like–CoVs in bats highlights the increasingly recognized importance of bats as reservoirs of emerging viruses (*36*). Moreover, the recent emergence of SARS-CoVs and other bat-associated viruses such as henipaviruses (*37**,**38*), Menangle, and Tioman viruses (*36*), and variants of rabies viruses and bat lyssaviruses (*38**,**39*) also supports the contention that viruses, especially RNA viruses, possess more risk than other pathogens for disease emergence in human and domestic mammals because of their higher mutation rates (*40*).
